# The Making of a Queen: TOR Pathway Is a Key Player in Diphenic Caste Development

**DOI:** 10.1371/journal.pone.0000509

**Published:** 2007-06-06

**Authors:** Avani Patel, M. Kim Fondrk, Osman Kaftanoglu, Christine Emore, Greg Hunt, Katy Frederick, Gro V. Amdam

**Affiliations:** 1 School of Life Sciences, Arizona State University, Tempe, Arizona, United States of America; 2 Entomology Department, Purdue University, West Lafayette, Indiana, United States of America; 3 Animal and Aquacultural Sciences Department, Norwegian University of Life Sciences, Aas, Norway; University of Texas Arlington, United States of America

## Abstract

**Background:**

Honey bees (*Apis mellifera*) provide a principal example of diphenic development. Excess feeding of female larvae results in queens (large reproductives). Moderate diet yields workers (small helpers). The signaling pathway that links provisioning to female developmental fate is not understood, yet we reasoned that it could include TOR (target of rapamycin), a nutrient- and energy-sensing kinase that controls organismal growth.

**Methodology/Principal Findings:**

Here, the role of *Apis mellifera* TOR (amTOR) in caste determination is examined by rapamycin/FK506 pharmacology and RNA interference (RNAi) gene knockdown. We show that in queen-destined larvae, the TOR inhibitor rapamycin induces the development of worker characters that are blocked by the antagonist FK506. Further, queen fate is associated with elevated activity of the *Apis mellifera* TOR encoding gene, *amTOR*, and *amTOR* gene knockdown blocks queen fate and results in individuals with worker morphology.

**Conclusions/Significance:**

A much-studied insect dimorphism, thereby, can be governed by the TOR pathway. Our results present the first evidence for a role of TOR in diphenic development, and suggest that adoption of this ancestral nutrient-sensing cascade is one evolutionary pathway for morphological caste differentiation in social insects.

## Introduction

TOR is the central component of a conserved eukaryotic signaling pathway that regulates cell and organismal growth in response to nutrient status [1], [2]. Growth rate correlates with ribosome number and metabolism, and TOR-dependent growth control in yeast and *Drosophila* involves transcriptional regulation of ribosomal and metabolic genes [3], [4]. Suppression of the *Drosophila* TOR pathway results in prolonged pre-adult development and reduces larval and adult body sizes [2].

In sum, these signatures of experimental variation in TOR signaling strikingly resemble the naturally occurring diphenism of the highly eusocial honey bee, where two alternative female phenotypes – the reproductive queen caste and the facultatively sterile worker caste – differentiate through social manipulation of larval nutrient status [5]. Queen-destined individuals, which receive a rich diet of royal jelly as larvae, are from the 3^rd^ instar characterized by accelerated larval growth, upregulation of larval ribosomal and metabolic gene expression, rapid pre-adult development and large body sizes [5]–[7]. Worker-destined larvae, which receive a moderate diet of less nutritious jelly [5], are characterized by the opposite of all of these. Moreover, honey bee queens and workers diverge in morphological characters, other than organismal size, that involve the differential growth of body parts ([5], see below).

This pattern led us to hypothesize that the evolution of caste diphenism in honey bees involved adoption of TOR signaling as an ancestral mechanism for regulation of phenotypic plasticity in response to variation in nutrient status. Consequently, honey bee caste determination was predicted to be amTOR-dependent, and queen vs. worker development to be conditional on high vs. low amTOR signaling in larvae, respectively.

Here, we first use pharmacology to implicate amTOR in the differentiation of female honey bees into queens and workers. Thereafter, we establish that *amTOR* is expressed at higher levels in queen-destined females. Finally, we make use of *amTOR* RNAi combined with double RNAi controls; *i)* exposure to GFP double stranded RNA and *ii)* knockdown of the honey bee *vitellogenin* gene; to demonstrate that queen-fate is blocked and workers develop when *amTOR* activity is reduced during development. This new insight represents the first evidence for a central role of TOR in a naturally occurring diphenism.

## Results

### The TOR inhibitor rapamycin induces worker characters in queen-destined individuals

We first exposed colony-reared queen- and worker-destined 4–5^th^ instar larvae to treatments with rapamycin, FK506 and vehicle control (2% ethanol). Pharmacological effects in younger stages could not be examined, as treated 1–3^rd^ instar larvae were rejected by colonies. Rapamycin and FK506 are structural analogues that compete for binding to the highly conserved FK binding protein-12 (FKBP-12). The rapamycin-FKBP-12 complex, but not the FK506-FKBP-12 complex, inhibits TOR activity and, thus, rapamycin-mediated interference with TOR signaling can be antagonized (competitively inhibited) by FK506. This approach has been used with high specificity to study TOR in insects [8].

**Figure 1:**
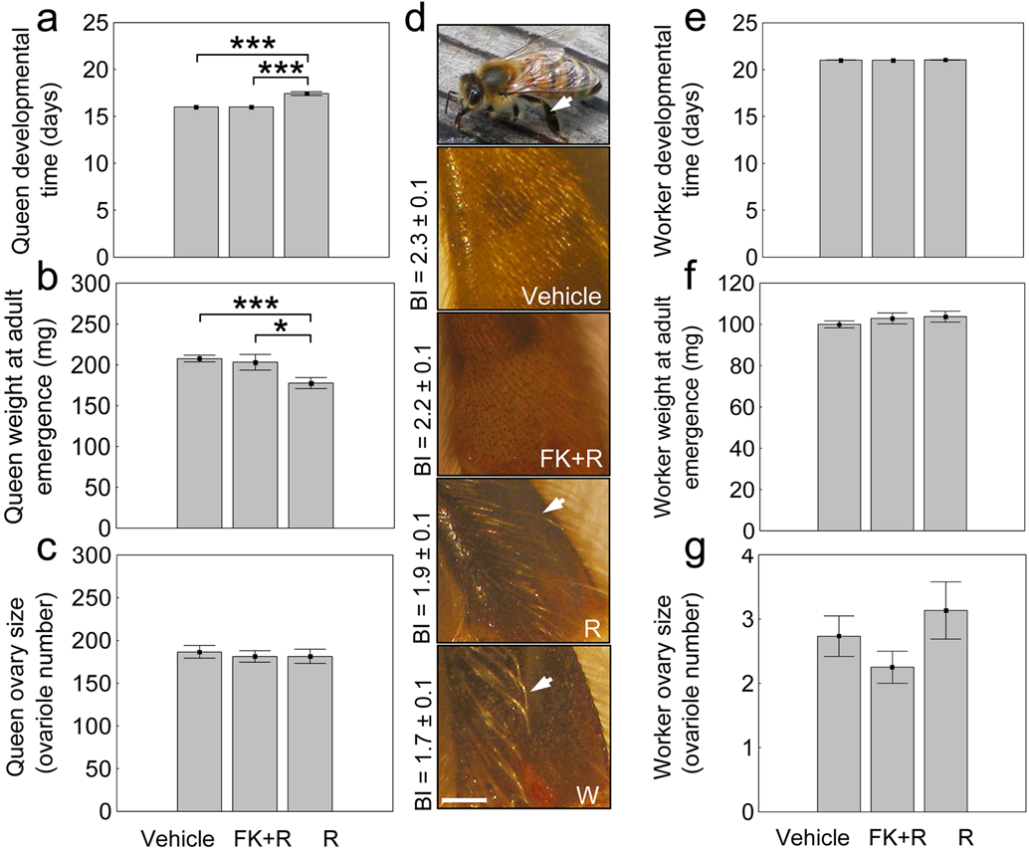
Effect of rapamycin/FK506 pharmacology on caste characters in honey bees (Vehicle = 2% ethanol in insect saline; FK+R = sequential treatment of FK506, rapamycin; R = rapamycin; W = worker as comparison). In queen-destined individuals, rapamycin: a, prolonged development and b, reduced wet-weight (size) at adult emergence; c, ovary size was not affected by pharmacology but rapamycin d, induced the worker-specific trait corbicula (arrow in top panel shows location on hind leg); pictures of hind leg basitarsus covered with short hairs in Vehicle and FK+R, and by long corbicular hairs (e.g. arrows) in R and W (scale bar 0.25 mm). Corbicula is associated with low basitarsus length/width ratio [9], and in accord R individuals showed a decrease in this Basitarsal Index (BI) mean±s. e. noted in d (ANOVA: *F*_3,21_ = 21.48, *P*<0.00001, Fisher LSD: *P*<0.00001; vs. between Vehicle and FK+R controls and between worker-destined groups *P*>0.50). e–g, rapamycin/FK506 pharmacology did not affect worker-destined bees. Bars are means±s. e. (asterisk *P*<0.02, two asterisks *P*<0.005, three asterisks *P*<1.0 10^−6^).

In queen-destined individuals, rapamycin prolonged pre-adult development (ANOVA: *F*_2,22_ = 66.0, *P*<0.0001, Fig. 1a), reduced wet-weight (size) at adult emergence (ANOVA: *F*_2,20_ = 5.75.0, *P*<0.01, Fig. 1b), and caused appearance of corbicula (pollen basket), a worker-specific morphological trait, while ovarian morphology remained queen-like (ANOVA: *F*_2,20_ = 0.17.0, *P* = 0.84, Fig. 1c–d; queens have much larger ovaries than workers). Nutritional manipulation of 4–5^th^ instar larvae, e.g., by transferring queen larvae into worker diet and *vice versa*, leads to similar intercastes with some traits characteristic of workers and some characteristic of queens (see [9] and refs. therein). Honey bee caste differentiation is a sequential process, with ovary size determined during the 3^rd^ larval instar, and corbicula during the 4–5^th^ larval instars [9]. In queen-destined larvae, the TOR inhibitor rapamycin caused developmental changes toward worker characters; predictably excluding a trait determined prior to the experiment (ovary size) and including a trait determined concomitant with rapamycin treatment (corbicula).

These data indicate that honey bee caste determination is influenced by the TOR pathway, at least in late sequential phases. Worker-destined individuals were not affected by rapamycin/FK506 pharmacology (ANOVA developmental time: *F*_2,117_ = 0.51, *P* = 0.51; wet-weight: *F*_2,41_ = 0.64, *P* = 0.53, ovariole number: *F*_2,43_ = 1.68, *P* = 0.20, Fig. 1e–g), consistent with the prediction that worker determination occurs when amTOR signaling is low in larvae.

### Queen fate is associated with elevated activity of the *Apis mellifera* TOR encoding gene, *amTOR*

**Figure 2:**
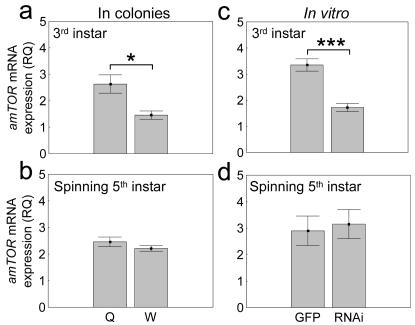
Relative quantities (RQ) of *amTOR* mRNA levels in honey bee larvae. In colonies a–b, queen- (Q) and worker-destined (W) larvae are characterised by a transient two-fold difference in *amTOR* expression (present in 3^rd^ instar larvae, absent in spinning 5^th^ instar larvae). *In vitro* c–d, RNAi was calibrated to decrease *amTOR* about two-fold in 3^rd^ instar knockdowns (RNAi) vs. controls exposed to GFP derived dsRNA (GFP). Suppression was transient, as often observed with RNAi. Thereby natural expression patterns of *amTOR* were mimicked (a–b vs. c–d). Bars are means±s. e. (asterisk *P*<0.01, three asterisks *P*<1.0 10^−6^).

Several mechanisms can influence TOR signaling [1], [2]; one is the transcript level of *TOR* mRNA [10]. As a next step, we explored if the critical decision-point of caste determination (3^rd^ larval instar) was characterized by variation in *amTOR* expression that correlated with developmental fate. Using larvae reared as queens and workers by colonies, we found that 3^rd^ instar queen-destined females had approximately two-fold higher *amTOR* mRNA levels than 3^rd^ instar worker-destined larvae (ANOVA: *F*_1,12_ = 9.46, *P*<0.01, Fig. 2a). Moreover, after larval feeding and growth were completed (spinning 5^th^ instar), this difference in *amTOR* expression was no longer present (ANOVA: *F*_1,11_ = 1.55, *P* = 0.24, Fig. 2b). Relative *amTOR* levels in larvae, therefore, are consistent with general patterns of growth [5] that characterize queen- and worker-destined individuals. Also, high vs. low *amTOR* activity in the 3^rd^ instar correlate with queen vs. worker developmental fate, respectively – in agreement with the role we propose for amTOR signaling in honey bee caste determination.

### *amTOR* gene knockdown blocks queen fate and results in workers

To test the effect of *amTOR* on caste development, we combined *in vitro* rearing of 1–5^th^ instar larvae (i.e., removed from the colony setting) with suppression of *amTOR* activity by RNAi. Larvae were given a diet made primarily of royal jelly that yields up to 50% queens. Due to methodological challenges [11], *in vitro* rearing typically does not yield a higher proportion of queens. The amount of *amTOR* double-stranded RNA (dsRNA) delivered to larvae was adjusted to yield a transient two-fold reduction of *amTOR* levels (Fig. 2c–d, 3^rd^ instar ANOVA: *F*_1,21_ = 32.06, *P*<0.00001, Fig. 2c, vs. spinning 5^th^ instar *F*_1,14_ = 0.11, *P*<0.75, Fig. 2d), thereby mimicking the relative expression pattern observed in colonies (Fig. 2a–b).

**Figure 3:**
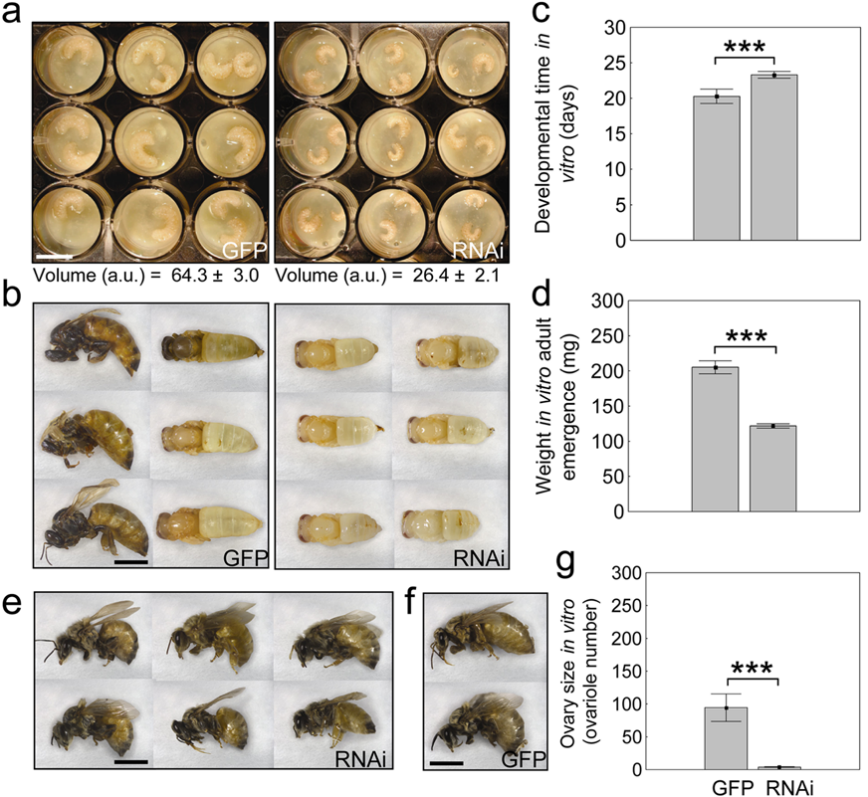
Effect of *amTOR* suppression on caste characters in honey bees. In comparison to control (GFP), *amTOR* RNAi (RNAi): a, reduced larval growth (exemplified by 4-day-old larvae in sections of a microtiter plate, larval volume is mean±s. e. arbitrary units a.u.); delayed development, b–c, where b is a snapshot of the full phenotypic variance in each group (individuals were 19 days old) – controls emerged with queen morphology, or had advanced pigmentation (indicates more rapid development) and large size. RNAi bees were lightly pigmented and small in size; the latter effect is shown also in d, the wet-weight at adult emergence and in e, the final adult size. Adult size did not overlap between treatment groups (e vs. f, where f shows the two control bees with lowest wet-weight: 155 and 161 mg; weight-range after RNAi was 108–136 mg). *amTOR* RNAi also reduced ovary size g, to ovariole numbers characteristic of workers (GFP range was 12–180, RNAi range was 2–7 ovarioles per ovary). In sum, the divergence between control and *amTOR* RNAi is characteristic of queen vs. worker development [5]. Bars are means±s. e. (three asterisks *P*<1.0 10^−3^). Scale bars a: 10 mm; b, e, f: 5 mm.

Initially, the same amount of dsRNA derived from green fluorescent protein (GFP) sequence was used as control. We found that suppression of *amTOR* activity reduced the growth of the developing larvae (ANOVA: *F*_1,28_ = 99.29, *P*<0.00001, Fig. 3a), prolonged pre-adult development (ANOVA: *F*_1,19_ = 48.00, *P*<0.00001, Fig. 3b–c), reduced wet-weight (size) at adult emergence (ANOVA: *F*_1,19_ = 68.28, *P*<0.00001, Fig. 3d), and ultimately caused all *amTOR* knockdowns (*n* = 10) to emerge with fully developed worker morphology (Fig. 3e). In the control group, the proportion of females with queen morphology was 55% (*n* = 11), as expected from diet alone. Remaining controls were intercastes (e.g., Fig. 3f–g), a result common with *in vitro* rearing [11]. Thus, in a nutritional environment that encouraged queen differentiation, a physiologically relevant suppression of *amTOR* activity was sufficient to silence queen development. Instead, individuals emerged with a worker morphotype.

**Figure 4:**
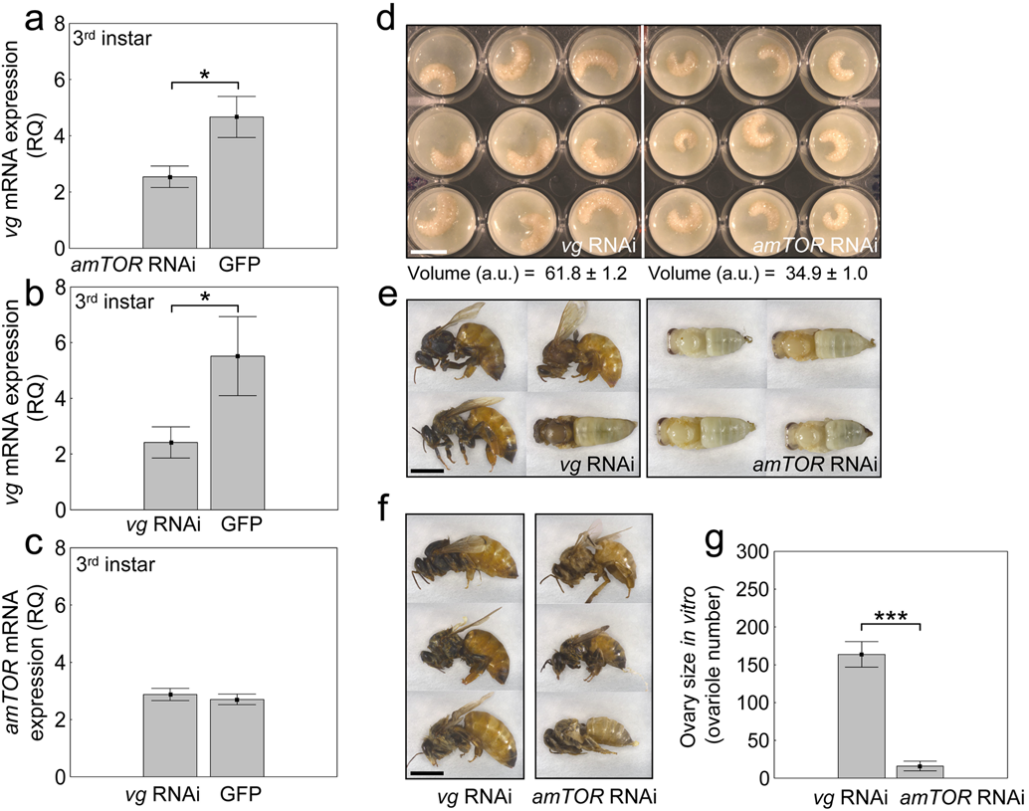
Control experiment comparing *amTOR* suppression (*amTOR* RNAi) with *vitellogenin* suppression (*vg* RNAi). In 3^rd^ instar larvae, *vitellogenin* mRNA a, was reduced by *amTOR* RNAi. Yet when b, *vitellogenin* was similarly suppressed by *vg* RNAi, *amTOR* c, remained unaffected. Thus, *vg* RNAi is an unconfounded control for *amTOR* RNAi. In comparison to *vg* RNAi, *amTOR* RNAi: d, reduced larval growth (exemplified by 5-day-old larvae, larval volume is mean±s. e., arbitrary units); and delayed development (days until adult emergence: *vg* RNAi, 20.14±0.14; *amTOR* RNAi, 24.00±0.58) – also shown in e, a snapshot of phenotypic variance (20-day-olds), which demonstrates that *vg* RNAi bees had emerged with queen characters or had advanced pupal pigmentation; while *amTOR* RNAi bees were pupae, lightly pigmented and small. f, adult size of the last *vg* RNAi bees to emerge (21^st^ day) *vs.* TOR RNAi bees (adult wet-weight mg: *vg* RNAi, 197.14±7.94; *amTOR* RNAi 119.67±12.03). In comparison to *vg* RNAi, *amTOR* RNAi also reduced ovary size g, (range *vg* RNAi: 62–210; *amTOR* RNAi: 4–26 ovarioles/ovary, ANOVA: *F*_1,8_ = 118.92, *P*<0.00001). These data accurately replicate our comparison of GFP controls and *amTOR* RNAi (Fig. 3). Bars are means±s. e. (asterisk *P*<0.05). Scale bars d: 10 mm; e, f: 5 mm.

The specificity of the effect of *amTOR* was tested in a control experiment where the gene *vitellogenin* was suppressed by RNAi. *vitellogenin* is transcribed in male and female honey bee larvae, and the protein is present in low amounts during larval development [12]. In adults, vitellogenin is a major yolk precursor with pleiotropic effects [13], [14]. Putative roles in larvae remain unclear, but previous experiments show that *vitellogenin* knockdown does not obstruct normal development [15], [16], suggesting that *vitellogenin* could serve as a valid RNAi control. Also, we verified that although *amTOR* RNAi reduces *vitellogenin* mRNA levels (ANOVA: *F*_1,26_ = 6.07, *P*<0.02, Fig. 4a) *vitellogenin* RNAi does not affect *amTOR* (for *vitellogenin* ANOVA: *F*_1,15_ = 5.25 *P*<0.04, Fig. 4b, vs. for *amTOR F*_1,41_ = 0.36, *P* = 0.55, Fig. 4c). This information is consistent with a positive effect of TOR on *vitellogenin* transcription described in mosquito [8], and further shows that *vitellogenin* RNAi is not confounded by effects on *amTOR*.

In comparison to *vitellogenin* RNAi, *amTOR* RNAi reduced larval growth (ANOVA: *F*_1,34_ = 299.20, *P*<0.0001, Fig. 4d); delayed development (ANOVA: *F*_1,8_ = 87.48, *P*<0.0005, Fig. 4e), and reduced final adult size (ANOVA: *F*_1,8_ = 28.67, *P*<0.001). As observed for GFP dsRNA control (Fig. 3), *vitellogenin* knockdowns emerged with queen morphology; *amTOR* knockdowns emerged as workers (Fig. 4f–g). Thus, the blocking of queen fate that occurs after *amTOR* knockdown is not an artifact of RNAi.

## Discussion

Our findings show that *amTOR* can govern the suites of morphological characters that differentiate honey bee queens and workers. As a premier example of diphenic development, these phenotypes have been subject to intense study over the last several decades [6], [7], [17], [18]. The association between nutrition and developmental fate has been recognized for more than 100 years [19], but the gene regulatory link between nutrient status and caste determination has remained unidentified. Therefore, the insight that amTOR signaling can regulate the honey bee queen-worker dimorphism provides the first evidence for an answer to this much studied question in developmental biology.

Knowledge is limited on how nutrition affects TOR and how TOR influences naturally occurring phenotypic plasticity in higher eukaryotes [1], [2], [20]. Our results suggest that the intersection between larval nutrition and *amTOR* activity, and between larval amTOR signaling, growth, size and developmental fate, are useful systems for obtaining such insights. Through crosstalk, TOR action can be conditional on insulin/insulin-like signaling (IIS), a major growth factor signaling pathway that regulates growth and energy storage [21]. IIS influences hormonal integration of growth partly as an upstream effector of juvenile hormone in *Drosophila* [22]. Some genes of the IIS pathway are upregulated in honey bee queen larvae [18], but a causal connection to caste development remains to be established. Yet prior work points to juvenile hormone as an endocrine integrator of caste differentiation during late larval instars in honey bees [17], [23]. Caste development, therefore, is likely to involve interaction between amTOR, IIS and juvenile hormone signaling.

The conserved role of TOR in growth control also suggests that our findings have general implications for the understanding of insect social evolution. Evolution of sociality in hymenopteran insects likely relied on the genetic assimilation of facultative regulatory architectures that affected female fecundity (e.g., adoption of gene networks involved in nutritional signaling and reproductive hormonal pleiotropy [24], [25]). A bias toward worker behavior (maternal care directed at siblings) was achieved by curtailing the reproductive capability of some offspring, which then remain at the natal nest. In many insect taxa, body size and fecundity are correlated in females [26], [27]. This relationship is positively affected by larval nutrition [28], and is likely ancestral in hymenopterans [26]. Thus, from a primitive social setting (without castes), developmental plasticity to scale size and fecundity through nutrition could be adopted to produce offspring with different reproductive capacities (reproductive castes). In accord with this reasoning, the most basal morphological specialization in social hymenopterans is an increase in size of queens or a decrease in size of workers [29]. Combined with the results presented here, these perspectives suggest that the TOR pathway was one preadaptation that enabled morphological castes – and thus advanced eusocial life – to evolve in insects.

## Materials and Methods

### Rapamycin/FK506 pharmacology

Rapamycin and FK506 (Sigma-Aldrich) were dissolved in 96% ethanol (5 mg/ml and 15 mg/ml stock, respectively), stored at −20°C and diluted as fresh aliquots in insect saline to 2% ethanol vehicle. Sister honey bee larvae from two colonies were cofostered as queens and as workers using standard apicultural procedures. Eggs were obtained from queens caged for 6 h to control inter-individual variation in age (±3 h). Larvae were retrieved from their colonies every 12 h, and rapamycin, FK506, FK506 + rapamycin, or vehicle control was pipetted into their natural diet (larvae lie in cells, immersed in food). Doses were adjusted to average larval weight [30]; 0.02 vs. 0.06 µg/mg for rapamycin and FK506, respectively. Larvae were subsequently kept in an incubator (33°C, 80% RH) for 40 minutes, and then returned to the colonies. For FK506 + rapamycin treatment, FK506 was given first and rapamycin 20 min later (returning larvae 20 vs. 40 min after rapamycin treatment did not affect results; FK506 alone did not affect caste characters, data not shown). All treatments triggered extensive rejection of larvae when 1–3^rd^ instars were exposed (>85% removal). Older honey bee larvae are less likely rejected [31], and a higher percentage survived (mortality was 24%) when treatments were initiated during the 4^th^ instar. Bees treated as 4–5^th^ instar larvae were collected at adult emergence, weighed and examined for key morphological characters that distinguish workers from queens [5], [9]: ovary size (worker 2–16, queen >100 ovarian filaments per ovary) and corbicula (present in worker, absent in queen). For details on protocols for quantification of ovary size, see Dedej et al. [9].

### Additional morphological characters

Information was obtained also on notched mandibles and smooth stinger (present in queen, absent in worker). Detailed data on these morphological characters are not shown, as their occurrence was in general agreement with the two distinguishing queen characters that we report: *i)* ovary size with >100 ovarioles/ovary; and *ii)* the absence of corbicula (pollen basket).

### *amTOR* expression in queen- and worker destined larvae

Larvae were staged and reared as queens and as workers as described above. At collection, 3^rd^ instar was verified by measuring the head-capsule width of a subset of the larvae [30]. Spinning 5^th^ instar larvae were collected while spinning. Larvae were transferred to TRIzol reagent (Invitrogen) and stored (−80°C). RNA was extracted as described previously [32], except for an additional chloroform extraction step. Primers were derived from *amTOR* sequence (XM 625127): 5′-AACAACTGTTGCTGACGGTG-3′, 5′-GTTGCAGTCCAGGCTTTTTG-3′. Individual *amTOR* mRNA levels were determined by real-time RT-PCR in triplicate (ABI Prism 7500 Applied Biosystems), and analyzed using the comparative CT method with actin as housekeeping gene [32].

### *amTOR* RNAi

*amTOR* cDNA was partially cloned (bp 5430–6871) and used as template in PCR. Primers were fused with T7 promoter sequence (underlined) 5′-TAATACGACTCACTATAGGGCGATAGTCCGGGACAGCCAATAG-3′, 5′-TAATACGACTCACTATAGGGCGACGATCACCAAGACCAAGGAT-3′. *amTOR* dsRNA (bp 6129–6713, i.e., 585 bp in length) and dsRNA derived from GFP encoding sequence (503 bp) was synthesized as described before [33]. First instar larva were collected from two different colonies, grafted into a pool of V.S. diet [34] (see also “Diets for *in vitro* rearing” below), and transferred to an incubator (33°C, 80% RH). The next day, larvae were grafted into 24-well microtiter plates with fresh V.S. diet with 150 µg/ml dsRNA (protocol modified after [34]). Diet with dsRNA was replenished twice daily for two days. Then, V.S. diet was substituted with D1 diet without dsRNA for two days, and finally D1 diet was spiked with 30% v/v 12/12 diet until larvae defecated prior to pupation. Defecating larvae were transferred to empty 24-well microtiter plates and left to pupate and emerge over 16% H_2_SO_4_ (33°C, 80% RH, mortality was about 50%)

### *vitellogenin* RNAi

The RNAi protocol was identical to the one outlined for *amTOR*, except that the amount of dsRNA was adjusted to 80 µg/ml diet. This quantity suppressed *vitellogenin* mRNA levels in 3^rd^ instar larvae to approximately the same extent as *amTOR* RNAi. Mortality was about 50%.

### Diets for *in vitro* rearing

V.S. diet [35]: 50% fresh-frozen commercial royal jelly, 6% glucose, 6% fructose, 1% yeast extract and 37% d-H_2_0; D-1 diet: 53% fresh-frozen commercial royal jelly, 6% glucose, 6% fructose, 1% yeast extract and 34% d-H_2_0; 12/12 diet: 53% fresh-frozen commercial royal jelly, 12% glucose, 12% fructose, 1% yeast extract and 22% d-H_2_0. Diet preparation: glucose, fructose and yeast extract were dissolved in d-H_2_0. Freshly thawed royal jelly was added and the diet mixed well.

### Quantification of larval volume

Larval volumes were quantified from 16-bit grayscale pictures using the Quantity One volume analysis tool (Bio-Rad).

### Statistics

One-way ANOVA was used for the analyses. The datasets conformed to the assumptions of ANOVA, as determined by Bartlett's and Levene's tests. For data on pharmacology, LSD post hoc tests were used to identify the treatments that contributed to significant overall effects. Analyses were performed with Statistica 6.0.
